# Investigating the Electrical Properties of Different Cochlear Implants

**DOI:** 10.1097/MAO.0000000000002861

**Published:** 2020-09-15

**Authors:** Nol Swaddiwudhipong, Chen Jiang, Thomas G. Landry, Manohar Bance

**Affiliations:** ∗ School of Clinical Medicine; † Department of Clinical Neurosciences, University of Cambridge, Cambridge, UK; ‡ Division of Otolaryngology, Nova Scotia Health Authority, Halifax, Nova Scotia, Canada

**Keywords:** Cochlear implant, Electrode array configuration, Impedance spectroscopy, Transimpedance

## Abstract

This study characterises and compares electrical properties and current spread across four different makes of cochlear implants with differing electrode designs using a 3D-printed artificial cochlear model.

**Background:**

Cochlear implants are currently limited by current spread within the cochlea, which causes low spectral resolution of auditory nerve stimulation. Different cochlear implant makes vary in electrode size, shape, number, and configuration. How these differences affect cochlear implant current spread and function is not well known.

**Method:**

Each cochlear implant was inserted into a linear cochlear model containing recording electrodes along its length. Biphasic monopolar stimulation of each implant electrode was carried out, and the resultant waveform and transimpedance matrix (TIM) data obtained from the recording electrodes. This was repeated with each implant rotated 180 degrees in the cochlea model to examine the effects of electrode orientation. Impedance spectroscopy was also carried out at the apex, middle, and base of the model.

**Results:**

The four cochlear implants displayed similar TIM profiles and waveforms. One hundred eighty degrees rotation of each cochlear implant made little difference to the TIM profiles. Impedance spectroscopy demonstrated broad similarities in amplitude and phase across the implants, but exhibited differences in certain electrical parameters.

**Conclusion:**

Implants with different designs demonstrate similar electrical performance, regardless of electrode size and spacing or electrode array dimension. In addition, rotatory maneuvers during cochlear implantation surgery are unlikely to change implant impedance properties.

Cochlear implants (CIs) enable auditory perception in those with severe to profound hearing loss. CIs contain an electrode array which is surgically implanted into the cochlea. Sounds are externally detected, digitized, and processed into separate spectral bands. After transmission wirelessly across the skin, the envelope of the signal in these bands is presented via (usually) biphasic carrier pulses to the implanted array. Each frequency band is presented to a different electrode. The frequency bands assigned endeavor to follow the normal apical to basal tonotopic map in the cochlea, with lower frequency bands presented to electrodes placed more apically, and higher frequency bands to those placed more basally. These signals stimulate nearby auditory afferent nerve fibers.

Despite hardware and software improvements since they were first conceived, CIs currently offer auditory perception that is very limited in quality compared with normal hearing. One key limitation is poor spectral resolution of auditory nerve electrical stimulation, caused mainly by current spread in the cochlea during implant electrode activation. This means spectral channels “blur” together, limiting the number of effective spectral channels over which the CI can convey auditory information to nerve fibers. CI users struggle with music appreciation (1,2) and speech discrimination in noisy environments, which requires more spectral channels than are actually delivered by current implants (3). Indeed, the loss of spectral resolution in CIs contributes to poor speech recognition in challenging acoustic environments (4,5). Supporting this view, speech recognition improves with the number of implant electrodes activated, but plateaus after activating about seven electrodes representing just seven frequency bands, despite having up to 22 electrodes with theoretically the same number of available frequency bands (6). This suggests that the number of effective spectral channels does not increase with the number of electrodes beyond seven electrodes. It is likely that current spread causes diffuse stimulation of overlapping populations of auditory nerve fibers. This effect is compounded by the poor residual neural population in many deaf cochleas. However, it should be noted that some more recent studies show there is some improvement in speech perception when the number of active electrodes is increased beyond seven (7,8), suggesting that CI users may be able to use more spectral channels in some cases. Nevertheless, research continues to demonstrate poor spectral resolution in CIs, which can negatively impact speech intelligibility (9), suggesting that it remains a major limitation to sound quality.

Numerous electrical stimulation strategies have been proposed to focus current and improve spectral resolution by changing current source and sink configurations. These include bipolar and tripolar stimulation (10). Results have been mixed. There is evidence for better speech discrimination with partial tripolar stimulation compared with monopolar stimulation (11), but another study finds no difference in pitch discrimination between tripolar and monopolar stimulation (12). More recently, a modified form of tripolar stimulation termed dynamic current focusing is purported to give better spectral resolution under certain conditions (13). Another proposed strategy is phased array stimulation, which aims to produce focused electrical stimulation at a particular site by sending a complex phase-shifted array of inverted currents as the stimulation signal to all other sites that tries to cancel out the effects of current spread (14).

Successfully implementing these stimulation strategies relies on an understanding of the complex impedance and other electrical properties of CI stimulation within the cochlea, which influence stimulation waveforms and current spread. Electrical measurements have previously been carried out in in-vitro settings and CI users (15). Computational models have also been developed to analyze current spread in CIs (16,17).

The current availability of different makes of CIs increases the complexity of parameters. Different CIs vary in terms of electrode number, shape, size, spacing, configuration, and electrode array length. A study demonstrated that CI design and electrode configuration influenced the activation threshold and spectral pattern of activation (18). Another study suggested that differences in the electrical parameters of CIs may influence speech recognition (19). There has been little investigation into how design parameters that vary between different manufacturers affect the electrical properties and functional output of CIs. This prompts the need for a systematic comparison of the electrical properties of different CIs.

Another compounding factor is that electrodes are inserted by hand by different surgeons in cochleas that vary in size across the population. Hence, electrode orientation relative to the auditory nerve may vary from cochlea to cochlea even for the same electrode design, and we also wish to examine this effect. Another important factor, not studied in this paper, is the placement of electrodes relative to the neural population, i.e., lateral wall or perimodiolar.

In this study, we use an artificial cochlea model to measure the voltage generated by the stimulation of CIs of different designs, and examine the effects of the orientation of the CI electrodes in the model. Waveforms, transimpedance, and impedance spectroscopy results were compared between four CIs under relatively constant in-vitro conditions.

## MATERIALS AND METHODS

### Basic Setup

**FIG. 1:**
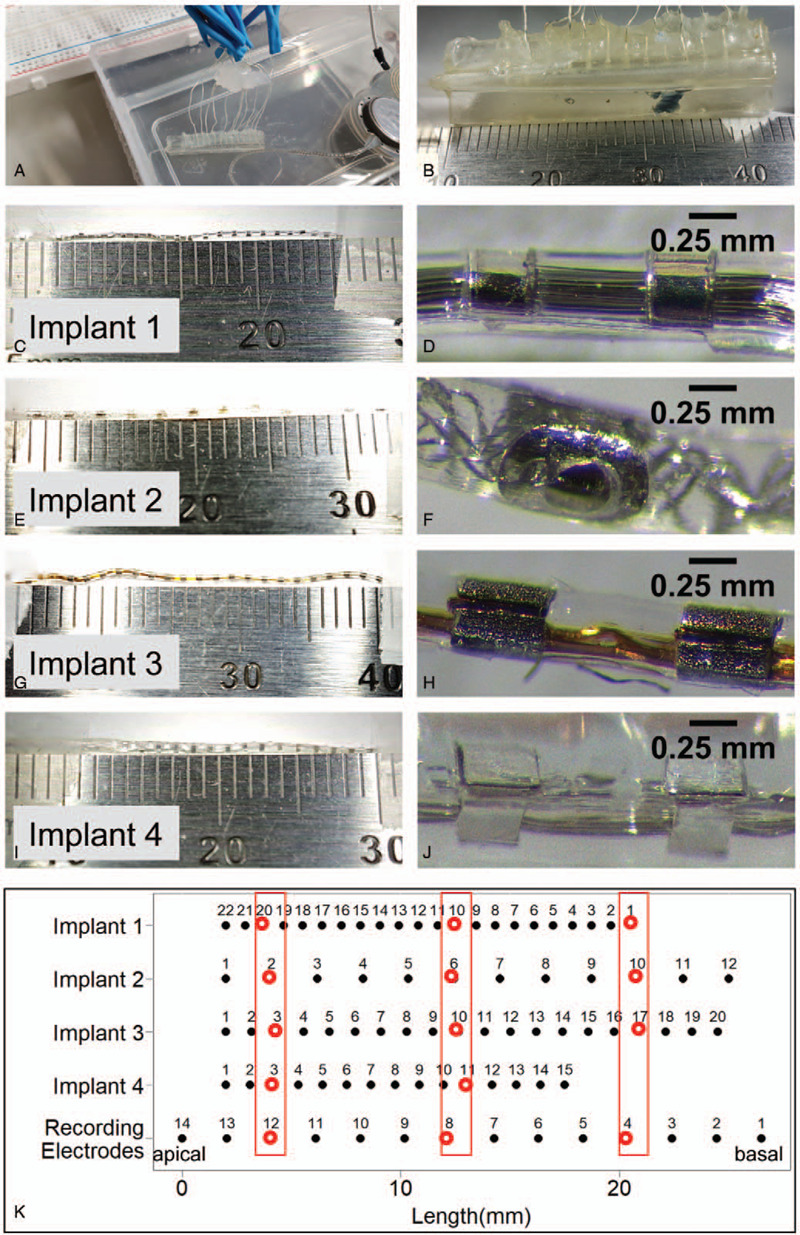
*A*, Cochlea model with recording electrodes and cochlear implant in situ immersed in 1% w/v NaCl solution. *B*, Cochlea model dimensions. *C*, Implant 1 length. *D*, Implant 1 with electrodes magnified. *E*, Implant 2 length. *F*, Implant 2 with electrodes magnified. *G*, Implant 3 length. *H*, Implant 3 with electrodes magnified. *I*, Implant 4 length. *J*, Implant 4 with electrodes magnified. *K*, Approximate position of the electrodes of each implant relative to the recording electrodes when each implant was inserted into the cochlea model. Red *boxes* and hollowed out *circles* indicate implant electrodes aligned with recording electrodes 4, 8, and 12 respectively.

In each in-vitro experiment (Fig. 1A), the CI was inserted into an artificial linear cochlea model (Fig. 1B), which was designed using Solidworks 2018 and 3D-printed with clear methacrylate resin using a Formlabs Form 2 3D printer. The lumen had a circular cross-section with a diameter varying along its length according to a previously published measurement of cross-sectional area in a human cochlea (20). Fourteen Teflon-coated silver wires (World Precision Instruments AGT1010) were inserted through the model wall every 2 mm, starting 1 mm from the basal opening, and affixed with manually applied UV-cured adhesive (Dymax Multi-Cure 9-911-REV-B). The entire setup was immersed in 1% w/v NaCl (Fisher Chemical, Loughborough, UK) solution, and experiments were carried out at room temperature. This basic in-vitro setup was common to all experiments.

Four CIs were tested. Each implant is referred to henceforth by their respective serial numbers as given below. Active stimulation length refers to the length of the electrode array from most apical to most basal electrode, inclusive of both end electrodes, as measured from photographs analyzed using ImageJ. The measurement for Implant 4 was extrapolated from the 15 electrodes present as one of the end electrodes was missing when the photo was taken.

Implant 1, the Slim Straight electrode array manufactured by Cochlear (Sydney, Australia), comprises 22 half-banded electrodes evenly spaced along its 19.1 mm active stimulation length (Fig. 1, C and D). Implant 2, the FLEX28 electrode array manufactured by MED-EL (Innsbruck, Austria), comprises 12 electrode sites evenly spaced along its 24.1 mm active stimulation length (Fig. 1, E and F). The first five sites apically each contains a half-banded electrode, while the remaining basal seven sites have a pair of half-banded electrodes at each site. In each of the basal pairs, the two electrodes are placed on opposite sides around the implant array. Implant 3, the EVO electrode array manufactured by Oticon (Smørum, Denmark), comprises 20 full-banded electrodes evenly spaced along its 23.1 mm active stimulation length (Fig. 1, G and H). Implant 4, the HiFocus 1j electrode array manufactured by Advanced Bionics (CA), comprises 16 half-banded electrodes evenly spaced along its 17.4 mm active stimulation length (Fig. 1, I and J). Due to the design of the electrode connections we had, stimulation for Implant 4 was only possible at electrodes 3, 5, 7, 9, 11, and 13.

In all experiments, each implant was placed in the cochlea model with the tip of its most apical electrode aligned with our recording electrode 13 in our cochlear model. The positions of implant electrodes relative to recording electrodes were numerically calculated and implant electrodes aligned close to recording electrodes 4, 8, and 12 respectively were identified for use as points of comparison between different implants (Fig. 1K).

### Transimpedance Matrix (TIM) Measurements

Transimpedance refers to the ratio of the voltage measured by each of the 14 recording electrodes in the cochlea model in response to stimulating current injected into one of the electrodes of an implant inserted into the cochlea model. This is repeated for all implant electrodes to build a TIM profile. All circuits were common grounded, i.e., all electrodes apart from the one being stimulated were used as common current return electrodes.

At each electrode on an implant, 14 current pulses were given sequentially. These current pulses were biphasic monopolar pulses (800 μA in amplitude, each phase lasting 32 μs). With each current pulse, we measured the voltage change over time sequentially at each of the 14 electrodes recording from within the cochlea model. Voltage was recorded at a 1 GHz sampling rate by a mixed signal oscilloscope (Teledyne Lecroy HDO4054A-MS) which conveyed the data into a PC with custom software implemented in LabView. For each recording, the waveform was saved and a peak-to-peak measurement calculated by subtracting the minimum from the maximum voltage recorded. This peak-to-peak measurement was divided by 800 μA to give the transimpedance. These set of steps were conducted at least three times for each implant and the resultant data averaged.

The above steps were repeated for each CI with the implant manually rotated 180 degrees within the cochlea model. Rotation was verified with reference to an ink mark dotted on the CI where it just extended out of the cochlea model. As above, at least three transimpedance measurements were made for each data point and the resultant data averaged.

### Impedance Spectroscopy

Impedance spectroscopy was carried out at the apex, middle, and base of each implant. For each CI, the implant electrodes close to recording electrodes 4, 8, and 12 respectively were determined (Fig. 1K). However, for Implant 1, data from stimulation of electrode 21 rather than 20 was used because of damage to electrode 20 before this set of experiments.

Each of these designated implant electrodes were stimulated with sinusoid current pulses over a frequency range of 10 Hz to 100 kHz with logarithmic intervals of 10 frequencies per decade, generated by a Precision inductance-capacitance-resistance (LCR) meter (LCR-6100 RS PRO). Voltage measurements taken from the corresponding recording electrode (4, 8, or 12) were sent to the LCR meter, which varied current magnitude to keep the sine wave amplitude constant at 0.1 V. Data from the LCR meter was conveyed to a computer with custom-made software implemented in LabView. Stimulation was carried out at least three times for each electrode and the resultant data averaged. All circuits were common grounded.

### Data Analysis

#### Waveform Time Constant

Time constants for biphasic pulse waveforms were determined by applying the SSasymp() function in R individually to each of the three stepped voltage changes in each waveform. At least three waveforms were analyzed for each data point, which reflects the mean obtained from all time constant values thus derived.

#### Electrical Model

Model parameters were derived using ZView software (Scribner Associates, Inc., North Carolina, USA). The fittings included all data points from 10 Hz to 100 kHz. Complex fittings were applied using Calc-Modulus data weighting. Parameters were calculated via curve fitting of the data obtained in each individual frequency sweep and the mean was then taken of values thus obtained from repeats.

## RESULTS

### Implants Generate Similar TIM Profiles

**FIG. 2:**
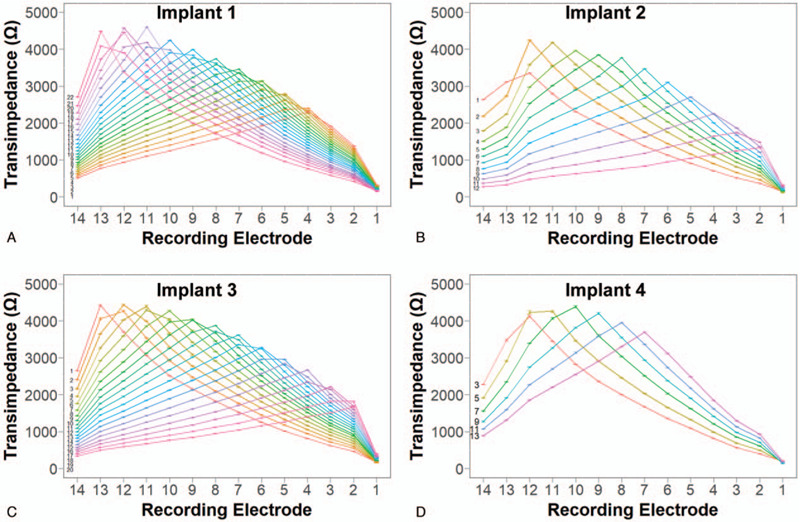
Complete transimpedance matrix profiles for each implant. Each line represents the mean transimpedance values recorded across the 14 recording electrodes when a specific implant electrode is stimulated, the number of which is labeled on the left. Error bars denote the standard deviation. *A*, Implant 1. *B*, Implant 2. *C*, Implant 3. *D*, Implant 4.

**FIG. 3:**
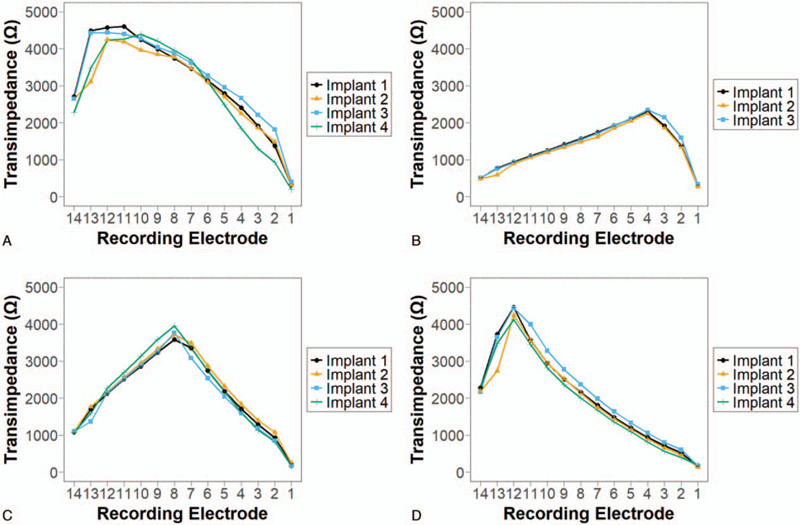
Comparison between transimpedance matrix profiles of different implants. *A*, Comparison of the maximum transimpedance value at each recording electrode in the respective implants. *B*–*D*, Mean transimpedance values at the 14 recording electrodes when a specific electrode on each implant is stimulated, with error bars denoting the standard deviation. *B*, The respective implant electrodes being stimulated lie close to recording electrode 4 (see Fig. 1K). *C*, Stimulation of implant electrodes close to recording electrode 8. *D*, Stimulation of implant electrodes close to recording electrode 12.

TIM profiles of the four implants agree well with one another (Fig. 2, A–D). Across the four implants, transimpedance values were higher at the apical end (recording electrodes 10–13) and showed a similar gradual decrease basally. The maxima measured at each recording electrode are similar across the four implants, following a decreasing trend from apex to base (Fig. 3A). However, slight differences are noted in the maxima. Implant 4 displayed reduced maxima at recording electrodes 2 to 5 and 13 to 14. These reduced values can be attributed to the lack of electrodes on Implant 4 available for stimulation at those locations. The reduced values recorded at those locations were the result of the stimulation of more distant implant electrodes.

Individual electrodes of each implant display similar TIM graphs compared with electrodes of other implants at a similar position in the cochlea model. Representative comparisons from the apex, middle, and base of the cochlea model exemplify this similarity. Figure 3B shows similar TIM profiles across Implants 1 to 3 when an electrode from each implant close to recording electrode 4 was stimulated (Fig. 1K). No nearby electrode from Implant 4 was available for stimulation. Figure 3C and D demonstrates the similarity in TIM profiles across all four implants when stimulating near recording electrodes 8 and 12 respectively.

### Implant Rotation Does Not Alter TIM Profile

**FIG. 4:**
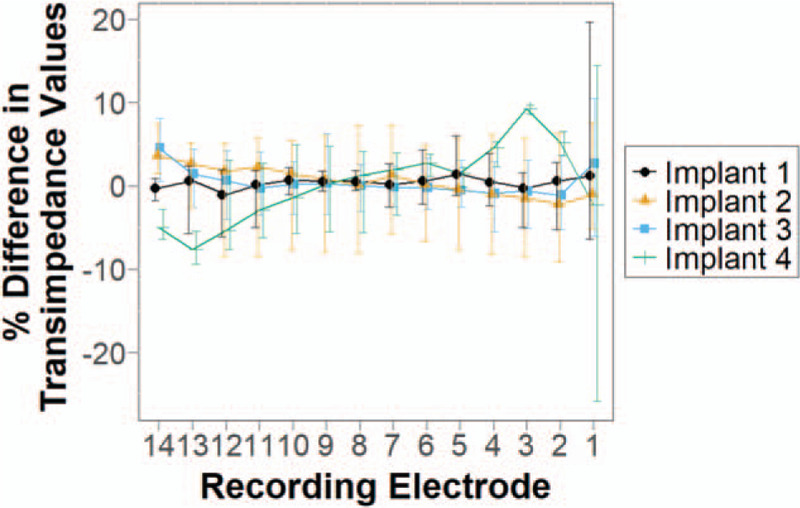
Comparison of transimpedances following 180 degrees rotation of each implant. Mean percentage difference of all transimpedance values at the respective recording electrodes before and after rotation, with error bars denoting the range of the percentage difference.

Each implant generated similar TIM profiles after 180 degrees rotation. In all four implants, the percentage difference between corresponding mean transimpedance values in the TIM graphs before and after rotation were close to 0% and rarely exceeded 10%. Figure 4 summarizes this data, showing the mean percentage difference between corresponding measurements at each of the recording electrodes, which rarely exceeded ±5%. There was no consistent increase or decrease in readings following rotation for any individual implant electrode stimulated. For all four implants, there was also no coherent global pattern to the differences observed following rotation.

Anomalies include the mean percentage difference exceeding ±5% for Implant 4 at recording electrodes 2 to 3 and 12 to 14, and a relatively wide range of percentage differences observed at recording electrode 1 for Implants 1 and 4. In all of these cases, there was no nearby implant electrode close to the respective recording electrodes available for stimulation because of the short length of these two implants. In addition, for Implant 4, stimulation was only possible at electrodes 3, 5, 7, 9, 11, and 13 due to breakages in the electrodes, and thus the length over which stimulation could be carried out was further shortened. The measurements taken were hence from the stimulation of more distant electrodes, which might have contributed to their increased variability. As the overall shape of the TIM profile is very similar across the four implants, it is highly unlikely that stimulating intervening electrodes would have revealed other aspects of voltage spread behavior.

### Implants Generate Similar Waveforms

**FIG. 5:**
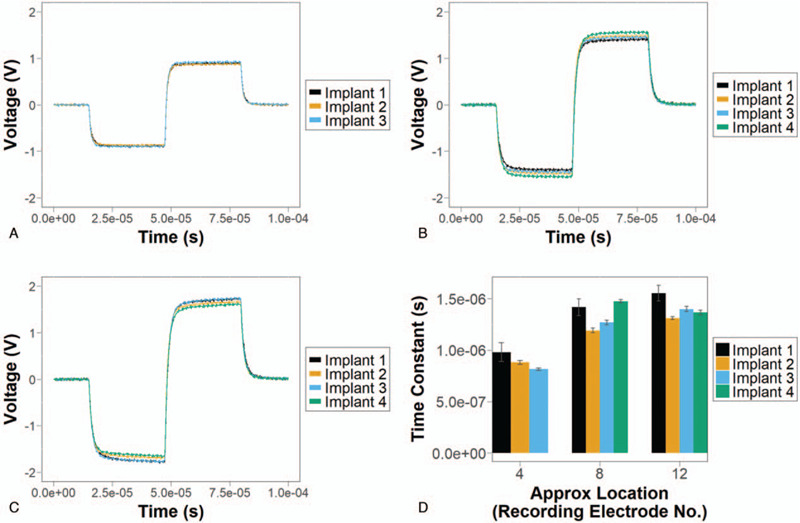
Comparison of waveforms between different implants. *A–C*, Examples of waveforms observed when stimulating an electrode on each implant close to recording electrodes 4, 8, and 12 respectively (see Fig. 1K). In *A*, implant 4 is not included as there were no implant electrodes close to recording electrode 4 due to the relatively shorter length of implant 4. *D*, Mean estimated time constant of voltage change when stimulating implant electrodes close to recording electrodes 4, 8, and 12, with error bars representing the standard deviation.

Waveforms generated during monopolar biphasic stimulation are similar across the four implants. Figure 5A shows examples of the similar waveforms generated by each implant during stimulation of an electrode aligned close to recording electrode 4 (Fig. 1K). Due to the shorter length of Implant 4, no implant electrodes close to recording electrode 4 were available for stimulation, and so Implant 4 is not included in Figure 5A. This similarity is also seen with implant electrodes aligned close to recording electrodes 8 and 12 (Fig. 5, B and C respectively). Time constants of voltage change calculated from the waveforms (see Methods, Data Analysis) agree well across the four implants for each of the locations in the cochlea model investigated (Fig. 5D).

### Impedance Spectroscopy

**FIG. 6:**
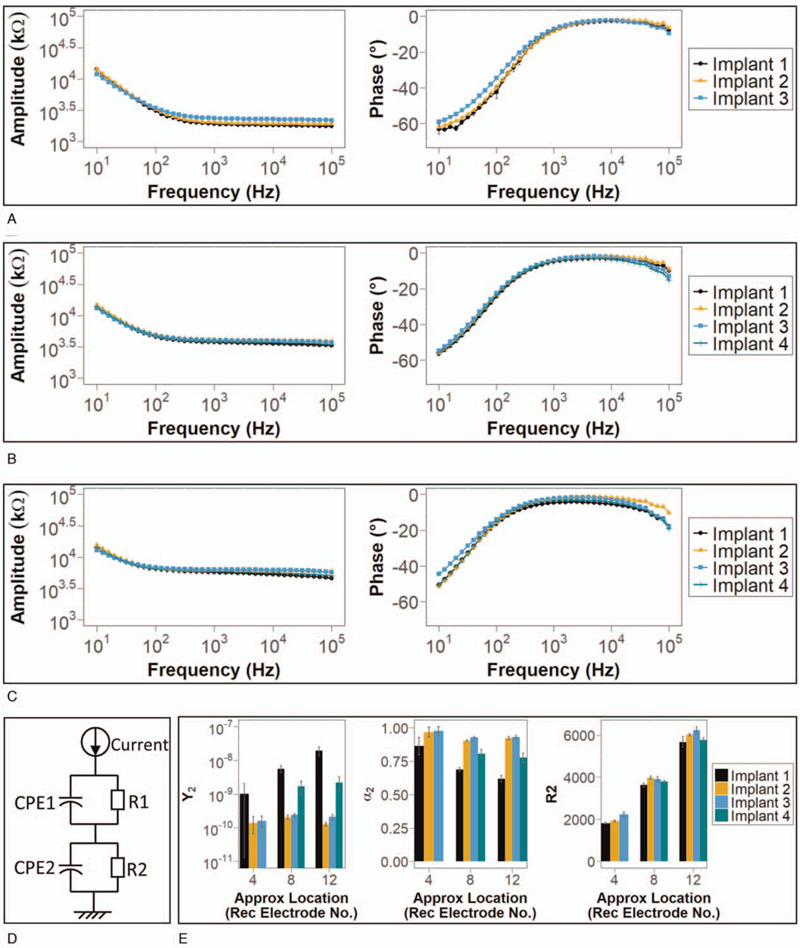
Comparison of impedance spectroscopy results between different implants. *A*–*C*, Amplitude and phase against frequency during sine-wave stimulation of an electrode on each implant close to recording electrodes 4, 8, and 12 respectively (see Fig. 1K, but electrode 21 is used in place of electrode 20 in Implant 1). In *A*, implant 4 is not included as there were no implant electrodes close to recording electrode 4 due to the relatively shorter length of implant 4. *D*, Electrical model of impedance in the cochlea model. E, derived electrical model parameters for each implant at different locations. All points and bars denote the mean while error bars represent the standard deviation. CPE indicates constant phase element; R, resistor; Rec, recording.

Impedance spectroscopy results were broadly similar across implants with minor differences observed. When stimulating an electrode on each implant aligned close to recording electrode 4, Implants 1 and 2 broadly agree in terms of phase and amplitude across all frequencies, while Implant 3 had slightly raised amplitude at higher frequencies and a less negative phase shift at lower frequencies (Fig. 6A). Due to the shorter length of Implant 4, no implant electrodes close to recording electrode 4 were available for stimulation, and so Implant 4 is not included in Figure 6A. Stimulation of implant electrodes aligned close to recording electrode 8 generated phase and amplitude graphs that were similar across the four implants. Slight differences in phase and amplitude were observed at high frequencies (Fig. 6B). At recording electrode 12, phase was similar but there was a less negative phase shift for Implant 2 at high frequencies and for Implant 3 at low frequencies (Fig. 6C). Amplitude was also similar among the four implants except for a decrease at higher frequencies in Implants 1 and 4. In Implant 1, electrode 21 was stimulated rather than electrode 20, which was damaged. As electrode 21 was further away from recording electrode 12, this might have contributed to the lower amplitude observed.

The parameters of an electrical model of impedance were calculated from the impedance spectroscopy results (see Methods, Data Analysis). The electrical model is based on a resistor in parallel with a constant phase element, then in series with another resistor in parallel with another constant phase element (Fig. 6D). The parameters calculated were the resistance values R1 and R2, and the *Y* and *α* terms that govern the impedance of the constant phase elements (CPE1 and 2) as per the equation:

$$Z_{CPE}=\frac{1}{Y\cdot {\left(j\omega \right)}^{\alpha }}$$

*α*_2_ and *Y*_2_ displayed some variation between the four implants. R2 was similar across all implants at the base, middle, and apex of the cochlea model and increased similarly in all implants from base to apex (Fig. 6E). R1 was much larger in magnitude than the impedance at CPE1 over the frequency range measured, and so the effect of CPE1 dominated in this part of the model. *α*_1_ and *Y*_1_ had similar values across the four implants (not shown).

## DISCUSSION

The similarity of TIM profiles suggests a similar pattern of current spread in all implants, showing that the variation in electrode array design across the four implants did not affect the spread of electrical stimulation per se. Current spread leads to poor spectral resolution even in implants with smaller and more numerous electrodes. In addition, the similarity in transimpedance values recorded across the four implants suggests that electrode array design did not significantly affect amplitude with the biphasic pulses used.

The similarity in waveforms between implants suggests that the different electrode array designs had little effect on the voltage signal measured at the recording electrodes, including amplitude and delay. Variation between time constants in the waveforms of different implants was on the order of 10^−7^ seconds. Such variation is likely biologically undetectable with negligible significance for implant functionality.

The similarity in waveforms are consistent with the theoretical prediction that different implant electrode designs would be expected to cause little difference in the cochlea except at the stimulating electrode-electrolyte interface. Beyond this interface, the amount and the rate of charges injected into the cochlea should be the same regardless of differences in electrode design. Voltage measurements were also conducted that showed very low current flow through the recording electrodes, and thus the effect of the electrode-electrolyte interface at the recording electrodes was negligible (21). Therefore, theoretically, voltage at the recording electrodes should be the same for different implants. The similarities in our results are thus consistent with our theoretical understanding.

The impedance spectroscopy experiments also demonstrated broad similarities across all implants. While slight differences were observed in amplitude and phase between different implants, there was no simple or consistent pattern in the differences. Notably, the differences between implants at each location are slight compared with the differences in the graphs of the same implant at different locations. From base to apex of the cochlea model, all implants exhibit an increase in amplitude especially over the higher frequencies, and a less negative phase shift especially over the lower frequencies. This shows that location along the cochlea model exerts a larger influence on phase and amplitude than electrode design of individual implants.

However, analysis of impedance spectroscopy data with our electrical model suggested possible differences between electrical properties of different implants. The pattern of variation of *Y*_2_ and *α*_2_ suggests a contributing effect of the implant electrode array. However, many factors might contribute, such as electrode size, material, and distance from recording electrodes. Future engineering work with more precise control over the variables involved will be required to elucidate the contributing factors.

Overall, these results show broad similarities between the four implants despite differences in electrode array design. The differences appear slight and have uncertain functional significance. This suggests that implants with different designs demonstrate similar electrical performance, regardless of electrode size and spacing or electrode array dimension. They also suggest that improvement in CI function is unlikely to be achieved solely by design changes like those encountered across the four implants investigated.

Of more immediate clinical relevance is the finding that 180 degrees rotation of the implants made little difference to the TIMs generated. Hence, surgical technique or rotatory maneuvers used to avoid damage to any remaining hearing are unlikely to affect implant performance. A suggestion for future work would be to control the distance between implant and recording electrodes to simulate differences in distance between implant electrodes and nerve endings in the modiolus.

The impact of these findings on speech perception for cochlear implant users is difficult to determine. What these results show is that the current spread is large, and not altered by the form factor of the electrodes. The degree of current spread has obvious implications for poor speech perception, as spectral cues in the speech envelope are not well resolved spatially. Our results would imply that there is likely to be little difference in speech recognition between the different types of electrodes if the coding strategy, insertion depth, and distance to neurones were similar. Because the current spread dominates so much of the signal reaching the neurones, even rotating the electrodes by 180 degrees has little effect on the electrical signal sensed at the distance we are measuring. This means that minor changes in the orientation of the electrode during insertion should not be of major consequence for hearing, unless they damage other structures such as the basilar membrane and cause loss of residual hearing. Of course, changing the distance to neurones, changing coding strategies, changing depth of insertion etc. may well have an effect, but this effect would have to be bigger than the current spread effect to have any practical impact on speech understanding.

The cochlea model used in this study has its limitations. Its linear structure is morphologically different to the spiral cochlea, and its material, temperature, and immersing solution all differ from in-vivo conditions. The results obtained hence reflect the electrical properties of the CIs in a simple artificial system, and do not take into account other physical and biological factors that may affect clinical outcome.

Nevertheless, the model offers the advantage over in-vivo recordings of providing impedance measurements close to the stimulating electrode. Impedance measurements in-vivo rely on stimulating implant electrodes themselves to act as recording electrodes. However, at the implant electrode being stimulated, electrical effects at the electrode-electrolyte interface distort the impedance measurement. Our system has dedicated recording electrodes that can measure impedance very close to the stimulating electrode, thus giving us an idea of impedance at the stimulating electrode, albeit in an in-vitro setting. This estimation of impedance may be useful in optimizing phased array and current steering stimulation strategies.

The model is also anatomically similar in base-to-apex diameter profile and length to the normal human cochlea, and represents a convenient and tractable means to compare the electrical properties of different CIs under constant conditions. A similar setup using a spiral-shaped cochlea model and materials and environmental controls that better simulate in-vivo conditions may prove useful in future studies. Such in-vitro studies, together with recently developed real-time in-vivo measurement techniques (22), may improve our understanding and enable engineering of implants with superior functionality.
